# Use of Germline *BRCA* Testing in Patients With Ovarian Cancer and Commercial Insurance

**DOI:** 10.1001/jamanetworkopen.2021.42703

**Published:** 2022-01-11

**Authors:** Stephanie Cham, Mary Beth Landrum, Nancy L. Keating, Joanne Armstrong, Alexi A. Wright

**Affiliations:** 1 Division of Gynecologic Oncology, Department of Obstetrics & Gynecology, Brigham and Women’s Hospital, Boston, Massachusetts; 2 Department of Health Care Policy, Harvard Medical School, Boston, Massachusetts; 3 Division of General Internal Medicine, Brigham and Women’s Hospital, Boston, Massachusetts; 4 Women’s Health and Genomics, CVS Health, Hartford, Connecticut; 5 Division of Population Sciences, Department of Medical Oncology, Dana-Farber Cancer Institute and Harvard Medical School, Boston, Massachusetts

## Abstract

This cross-sectional study evaluates changes in the rate of germline BRCA testing among patients with ovarian cancer between 2008 and 2018 and analyzes factors associated with testing rates.

## Introduction

Approximately 15% of patients with ovarian cancer have a germline *BRCA* (g*BRCA)* variation,^1^ which has important implications, including increased sensitivity to platinum-based chemotherapy and poly(ADP-ribose) polymerase inhibitors and improved survival.^2^ Testing first-degree relatives is also cost-effective cancer prevention.^3^ Since 2010, guidelines have recommended universal testing in ovarian cancer. However, testing rates are reportedly between 10% and 30%,^4^ and few studies have examined commercially insured populations or identified patient-, physician-, and practice-level characteristics associated with testing rates.

## Methods

Using data from a large national commercial insurer, this cross-sectional study included 12 989 patients with claims for ovarian, fallopian, or primary peritoneal cancers and a biopsy or surgery between 2008 and 2018. We restricted the cohort to patients with a biopsy or surgery and carboplatin or cisplatin within 6 months. We excluded those without surgery or outpatient visits, with less than 12 months of continuous insurance, with missing zip codes, or who were younger than 18 years (eFigure in the Supplement). We attributed patients to practices and physicians using outpatient evaluation and management claims with a diagnosis 6 months or less from the first outpatient claim for chemotherapy (eAppendix in the Supplement).^5^ The Harvard Medical School Committee on Human Studies deemed the study exempt from review and the requirement for informed consent because the study was a secondary analysis of previously collected data. We followed the Strengthening the Reporting of Observational Studies in Epidemiology (STROBE) reporting guideline for cohort studies.

The primary outcome was *gBRCA* testing using gene-specific and methodology-based procedure codes (eTable in the Supplement). Secondary outcomes included timeliness (ie, ≤6 months from biopsy/surgery) and median time from first chemotherapy claim to testing. We used χ^2^ tests and linear regression to assess patient, physician, and practice characteristics associated with outcomes. A 2-sided *P* ≤.05 was considered statistically significant. Analyses were performed using SAS statistical software version 14.1 (SAS Institute).

## Results

Among 3603 women with ovarian cancer (mean [SD] age, 57.0 [11.3] years), 1220 (33.9%) received g*BRCA* testing (Table). Testing rates increased from 14.7% (55 of 375 patients) in 2008 to 46.4% (96 of 207 patients) in 2018; the median time to testing decreased from 280.0 to 72.5 days (Figure). In adjusted analyses, testing was lower for older women (women ≥65 years vs <50 years: adjusted difference, −20.8 percentage points; 95% CI, −25.8 to −16.4 percentage points) and women with more comorbidities (Charlson Comorbidity Index score ≥2 vs 0: adjusted difference, −4.6 percentage points; 95% CI, −8.9 to −0.2 percentage points). Testing rates were similar among oncologists (medical vs gynecologic oncologist: adjusted difference, 1.5 percentage points; 95% CI, −1.8 to 4.7 percentage points) and lower in other physicians (other vs gynecologic oncologist: adjusted difference, −5.9 percentage points; 95% CI, −10.3 to −1.5 percentage points). Testing was higher at academic and NCI cancer centers compared with community practices (academic vs NCI: adjusted difference, 0.5 percentage points; 95% CI, −7.2 to 8.4 percentage points; community vs NCI: adjusted difference, −4.5 percentage points; 95% CI, −8.8 to −0.2 percentage points]). There was a statistically significant increase in testing over time (2018 vs 2008: adjusted difference, 32.0 percentage points; 95% CI, 24.4-39.7 percentage points), although rates remained below 50% for most years (Table). Results were similar for analyses of timeliness of *gBRCA* testing, which significantly improved from 2010 to 2018 (Table).

**Table.** Patient, Clinician, and Practice Characteristics Associated With Germline *BRCA* Testing Between 2008 and 2018

Characteristic	No. (%) (N = 3603)	*BRCA *testing				Time to testing			
		Unadjusted No. (%)	Unadjusted *P* value	Adjusted %	Adjusted difference (95% CI), percentage point	Unadjusted No. (%)	Unadjusted *P* value	Adjusted %	Adjusted % point difference (95% CI)
All, %	3603 (100)	1220 (33.9)	NA	NA	NA	867 (24.1)	NA	NA	NA
Patient									
Age, y									
<50	817 (22.7)	332 (40.6)	<.001	42.6	[Reference]	241 (29.4)	<.001	30.9	[Reference]
50-59	1345 (37.6)	515 (38.3)		39.3	−3.4 (−7.5 to 0.8)	366 (27.2)		27.6	−3.3 (−7.1 to 0.4)
60-64	656 (18.2)	225 (34.3)		36.0	−6.6 (−11.4 to −1.7)	159 (24.2)		25.2	−5.7 (−10.1 to −1.3)
≥65	785 (21.8)	148 (18.8)		21.8	−20.8 (−25.8 to −16.4)	101 (12.9)		14.4	−16.6 (−20.5 to −12.6)
Charlson comorbidity score									
0	2284 (63.5)	800 (35.0)	.01	36.6	[Reference]	569 (24.9)	.12	25.9	[Reference]
1	845 (23.5)	288 (34.1)		36.2	−0.4 (−4.0 to 3.2)	201 (23.8)		24.7	−1.1 (−4.4 to 2.1)
≥2	474 (13.2)	132 (27.8)		32.0	−4.6 (−8.9 to −0.2)	97 (20.5)		22.9	−2.9 (−6.9 to 1.0)
Education quartile, % HS or greater^a^									
First	903 (25.1)	270 (29.9)	.02	31.5	[Reference]	195 (21.6)	.06	22.2	[Reference]
Second	887 (24.4)	303 (34.2)		36.5	5.0 (0.2 to 9.7)	203 (22.9)		23.7	1.5 (−2.7 to 5.8)
Third	919 (25.5)	321 (34.9)		34.6	3.1 (−2.5 to 8.6)	231 (25.1)		24.2	2.2 (−2.9 to 7.1)
Fourth	894 (24.8)	326 (36.5)		37.1	5.6 (−0.8 to 11.9)	238 (26.6)		28.0	5.8 (0.1 to 11.6)
Income quartile, % <200% of the federal poverty level^a^									
First	901 (25.0)	323 (35.8)	.009	34.7	[Reference]	218 (24.2)	.006	21.8	[Reference]
Second	900 (25.0)	334 (37.1)		36.9	2.2 (−2.5 to 7.0)	253 (28.1)		27.6	5.8 (1.4 to 10.2)
Third	901 (25.0)	285 (31.6)		33.4	−1.3 (−6.9 to 4.2)	200 (22.2)		23.8	2.0 (−2.9 to 7.0)
Fourth	901 (25.0)	278 (30.9)		34.7	0.0 (−6.7 to 6.6)	196 (21.8)		24.7	2.9 (−3.0 to 8.9)
Region									
West	539 (15.0)	191 (35.4)	.77	35.7	[Reference]	140 (26.0)	.69	25.1	[Reference]
Midwest	572 (15.9)	187 (32.6)		34.4	−1.2 (−6.7 to 4.1)	137 (24.0)		24.9	−0.2 (−5.1 to 4.8)
Northeast	1004 (27.9)	344 (34.3)		34.3	−1.3 (−6.5 to 3.7)	242 (24.1)		24.0	−1.0 (−5.7 to 3.6)
South	1488 (41.3)	498 (33.5)		35.2	−0.5 (−5.1 to 4.1)	348 (23.4)		24.1	−0.9 (−5.1 to 3.2)
Location									
Urban	1716 (47.6)	579 (33.7)	.37	35.7	[Reference]	419 (24.4)	.13	26.2	[Reference]
Suburban	1731 (48.0)	596 (34.4)		35.4	−0.3 (−3.5 to 3.0)	421 (24.3)		25.6	−0.6 (−3.6 to 2.3)
Rural	156 (4.3)	45 (28.8)		33.8	−1.9 (−9.1 to 5.3)	27 (17.3)		21.7	−4.5 (−10.5 to 1.5)
Physician specialty									
Gynecologic oncologist	1542 (42.8)	543 (35.2)	.03	36.4	[Reference]	365 (23.6)	.08	24.1	[Reference]
Medical oncologist	1534 (42.6)	524 (34.2)		37.9	1.5 (−1.8 to 4.7)	392 (25.6)		27.9	3.9 (0.9 to 6.8)
Other physician	527 (14.6)	153 (29.0)		30.5	−5.9 (−10.3 to −1.5)	110 (20.9)		21.5	−2.6 (−6.5 to 1.4)
Practice type									
NCI cancer center	593 (16.5)	236 (39.8)	.002	36.2	[Reference]	168 (28.3)	.03	25.5	[Reference]
Academic	177 (4.9)	64 (36.2)		36.8	0.5 (−7.2 to 8.4)	44 (24.9)		25.4	0.0 (−7.0 to 7.0)
Community	2833 (78.6)	920 (32.5)		31.7	−4.5 (−8.8 to −0.2)	655 (23.1)		22.6	−2.8 (−6.8 to 1.1)
Year									
2008	375 (10.4)	55 (14.7)	<.001	12.6	[Reference]	23 (6.1)	<.001	3.7	[Reference]
2009	419 (11.6)	81 (20.8)		18.0	5.3 (0.2 to 10.5)	39 (9.3)		7.4	3.7 (0.0 to 7.5)
2010	458 (12.7)	119 (26.0)		25.5	12.9 (7.6 to 18.1)	78 (17.0)		15.6	11.9 (7.7 to 16.2)
2011	381 (10.6)	116 (30.4)		30.9	18.3 (12.5 to 24.1)	87 (22.8)		22.3	18.6 (13.8 to 23.5)
2012	365 (10.1)	121 (33.1)		32.7	20.1 (14.2 to 26.0)	92 (25.2)		23.9	20.2 (15.2 to 25.3)
2013	311 (8.6)	108 (34.7)		32.5	19.9 (13.6 to 26.3)	74 (23.8)		21.2	17.5 (12.2 to 22.9)
2014	290 (8.0)	116 (40.0)		37.6	24.9 (18.3 to 31.6)	77 (26.6)		24.0	20.4 (14.7 to 26.0)
2015	284 (7.9)	134 (47.2)		45.9	33.3 (26.5 to 40.0)	101 (35.6)		33.6	29.9 (23.9 to 35.9)
2016	294 (8.2)	159 (54.1)		53.2	40.6 (33.8 to 47.3)	116 (39.5)		37.9	34.2 (28.1 to 40.3)
2017	219 (6.1)	115 (52.5)		50.6	38.0 (30.5 to 45.4)	98 (44.7)		42.5	38.8 (31.8 to 45.8)
2018	207 (5.7)	96 (46.4)		44.7	32.0 (24.4 to 39.7)	82 (39.6)		37.6	33.9 (26.8 to 41.0)

Abbreviations: HS, high school; NA, not applicable; NCI, National Cancer Institute.

^a^ Census division and zip code–level measures used to identify proportion of residents who graduated from high school or above and the proportion of residents living in poverty.

**Figure.**
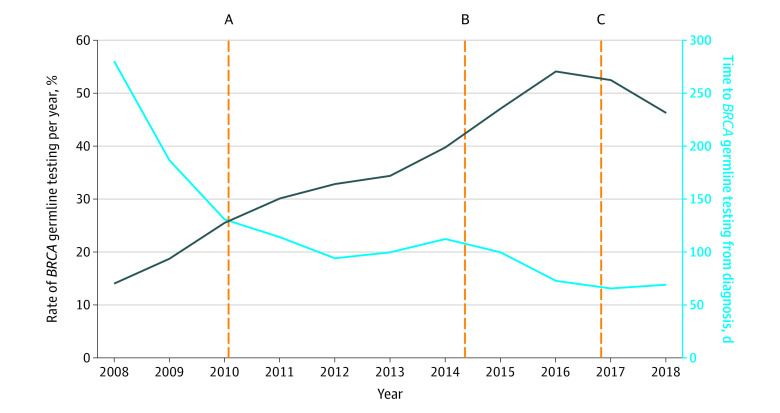
Rate and Days to Testing From Diagnosis of Germline *BRCA* Testing Annually Between 2008 and 2018 Vertical lines indicate landmark events related to germline *BRCA* testing. A, In 2010, National Comprehensive Cancer Network guidelines recommend universal genetic testing for all patients with ovarian cancer. B, In December 2014, the first poly(ADP-ribose) polymerase inhibitor receives US Food and Drug Administration approval with indication for treatment of recurrent disease in patients with germline *BRCA* who had received 3 or more prior lines of chemotherapy. C, On March 27, 2017, first poly(ADP-ribose) polymerase inhibitor receives US Food and Drug Administration approval for maintenance therapy in patients with platinum-sensitive recurrent ovarian cancer (independent of *BRCA*).

## Discussion

Despite unequivocal recommendations for universal genetic testing in ovarian cancer, only 33.9% of patients with commercial insurance were tested between 2008 and 2018—clear evidence it remains underused—and a minority received timely testing. In this study, medical and gynecologic oncologists had similar rates of testing, while other physicians tested less often, perhaps reflecting a lack of knowledge of guidelines. Nearly 80% of patients received care in community practices, where rates were statistically lower. Although independent practices often lack access to genetic counselors, women in this study had insurance coverage for in-person and telephonic counseling. Future studies should examine barriers to timely testing to identify scalable strategies for increasing testing, particularly for older women in community practices. Interventions targeting clinicians are essential because the absence of physician recommendations remains the largest barrier to testing.^6^ Study limitations include the use of biopsy/surgery for diagnosis date, limited sociodemographic characteristics, and the possibility that women received testing later.

## Notes

Supplement. **eFigure 1. **Flowchart of Cohort With Incident Diagnosis of Ovarian Cancer **eAppendix. **Assigning Patients to Practice and Physicians **eTable. **Codes Used for Cohort and Genetic Screening
