# Supplementary material for: Use of Germline BRCA Testing in Patients With Ovarian Cancer and Commercial Insurance
Source: JAMA Netw Open. 2022 Jan 11;5(1):e2142703. doi: 10.1001/jamanetworkopen.2021.42703 (PMC8753497; doi:10.1001/jamanetworkopen.2021.42703)
Supplement: Supplement. — eFigure 1. Flowchart of Cohort With Incident Diagnosis of Ovarian Cancer eAppendix. Assigning Patients to Practice and Physicians eTable. Codes Used for Cohort and Genetic Screening [file jamanetwopen-e2142703-s001.pdf]

Supplemental Online Content

Cham S, Landrum MB, Keating NL, Armstrong J, Wright AA. Use of germline *BRCA* testing in patients with ovarian cancer and commercial insurance. *JAMA Netw Open*. 2022;5(1):e2142703. doi:10.1001/jamanetworkopen.2021.42703

eFigure. Flowchart of Cohort With Incident Diagnosis of Ovarian Cancer
eAppendix. Assigning Patients to Practice and Physicians
eTable. Codes Used for Cohort and Genetic Screening

This supplemental material has been provided by the authors to give readers additional information about their work.

© 2022 Cham S et al. *JAMA Network Open.*

eFigure. Flowchart of Cohort With Incident Diagnosis of Ovarian Cancer

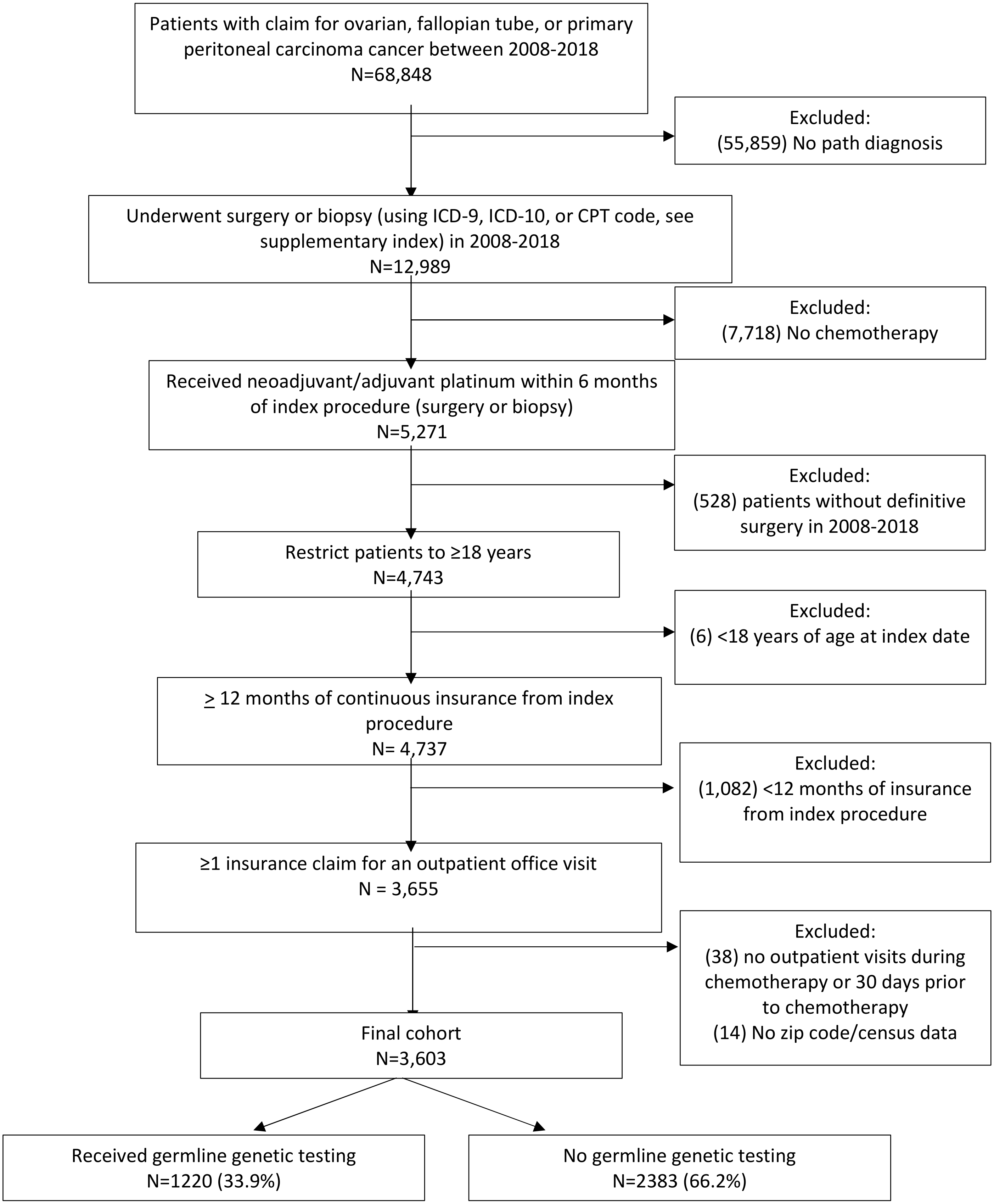

© 2022 Cham S et al. *JAMA Network Open.*

eAppendix. Assigning Patients to Practice and Physicians

We attributed patients to physicians based upon outpatient evaluation and management (E&M) claims with a cancer diagnosis within 6 months of the first outpatient claim for infused chemotherapy. Patients were attributed to practices according to the Tax Identification Number with the largest number of E&M visits within the first 6 months after the first chemotherapy claim. We assigned patients to physicians seen during these E&M visits according to the following categories based upon the variable “Servicing Provider Specialty” codes on the claim: gynecologic oncologists (20103, 2OGON), medical oncology (10311, 10315, 10316, 10309, 2ON or 2H). Patients were assigned to the practice and physicians with the most oncology visits in the 30 days prior to receipt of chemotherapy.

Patients without any visits to a medical or gynecological oncologist were assigned to the practice and physician with the most non-oncology visits. We attempted to match non-oncology physicians to a database of health systems, hospitals, and providers and recategorized physicians if this database indicated the physician specialty was medical or gynecological oncology.[1] The database includes data from the National Plan and Provider Enumeration System (NPPES), Medicare Data on Provider Practice and Specialty (MD-PPAS), Centers for Medicare & Medicaid Services (CMS) Physician Compare, IQVIA, and commercial claims that were combined to classify a specialty for each physician in the database. To code physician specialty uniformly, researchers at the National Bureau of Economic Research developed a physician specialty taxonomy based on board certifications offered by the American Board of Medical Specialties (ABMS) and mapped specialty classifications in each of the input data sets to the taxonomy.

[1] National Bureau of Economic Research. Health Systems and Provider Database. Accessed at https://www.nber.org/programs-projects/projects-and-centers/measuring-clinical-and-economic-outcomes-associated-delivery-systems/health-systems-and-provider-database-hspd-methodology-data-resources on March 24, 2021.

© 2022 Cham S et al. *JAMA Network Open.*

eTable. Codes Used for Cohort and Genetic Screening

**Ovarian, peritoneal, fallopian tube diagnosis codes**	
ICD-9 Codes	
Ovarian cancer and fallopian	183.x
Peritoneal cancer	158.8, 158.9
ICD-10 Code	
Ovarian cancer	C56.x
Fallopian tube cancer	C57.0x
Peritoneal cancer	C48.1, C48.2, C48.8
**Chemotherapy codes**	
J codes	
Carboplatin	J9045
Cisplatin	J9060, J9062
**Surgery codes**	
ICD-9 Code	
54.4	Omentectomy, excision, destruction peritoneal tissue
65.2	Wedge resection or partial excision of ovary
65.3x	Unilateral oophorectomy
65.4x	Bilateral oophorectomy
65.6x	Bilateral salpingo-oophorectomy
68.8	Pelvic exenteration
68.3-68.7, 68.9, 68.59	Hysterectomy
70.32	Excision/destruction cul de sac lesion
ICD-10 Code	
0D5U0ZZ	Destruction of Omentum, Open Approach
0D5U3ZZ	Destruction of Omentum, Percutaneous Approach
0D5U4ZZ	Destruction of Omentum, Percutaneous Endoscopic Approach
0UT04ZZ	Resection of Right Ovary, Percutaneous Endoscopic Approach
0UT14ZZ	Resection of Left Ovary, Percutaneous Endoscopic Approach
0UT54ZZ	Resection of Right Fallopian Tube, Percutaneous Endoscopic Approach
0UT64ZZ	Resection of Left Fallopian Tube, Percutaneous Endoscopic Approach
0UT20ZZ	Resection of Bilateral Ovaries, Open Approach
0UT27ZZ	Resection of Bilateral Ovaries, Via Natural or Artificial Opening

© 2022 Cham S et al. *JAMA Network Open.*

0UT28ZZ	Resection of Bilateral Ovaries, Via Natural or Artificial Opening Endoscopic
0UT2FZZ	Resection of Bilateral Ovaries, Via Natural or Artificial Opening With Percutaneous Endoscopic Assistance
0UT70ZZ	Resection of Bilateral Fallopian Tubes, Open Approach
0UT77ZZ	Resection of Bilateral Fallopian Tubes, Via Natural or Artificial Opening
0UT78ZZ	Resection of Bilateral Fallopian Tubes, Via Natural or Artificial Opening Endoscopic
0UT7FZZ	Resection of Bilateral Fallopian Tubes, Via Natural or Artificial Opening With Percutaneous Endoscopic Assistance
0UT94ZL	Resection of Uterus, Supracervical, Percutaneous Endoscopic Approach
0UT90ZL	Resection of Uterus, Supracervical, Open Approach
0UT90ZL	Resection of Uterus, Supracervical, Open Approach
0UT90ZZ	Resection of Uterus, Open Approach
0UT94ZZ	Resection of Uterus, Percutaneous Endoscopic Approach
0UT97ZZ	Resection of Uterus, Via Natural or Artificial Opening
0UT98ZZ	Resection of Uterus, Via Natural or Artificial Opening Endoscopic
0UTC0ZZ	Resection of Cervix, Open Approach
0UTC4ZZ	Resection of Cervix, Percutaneous Endoscopic Approach
0UTC7ZZ	Resection of Cervix, Via Natural or Artificial Opening
0UTC8ZZ	Resection of Cervix, Via Natural or Artificial Opening Endoscopic
0U5F0ZZ	Destruction of Cul-de-sac, Open Approach
0U5F3ZZ	Destruction of Cul-de-sac, Percutaneous Approach
0U5F4ZZ	Destruction of Cul-de-sac, Percutaneous Endoscopic Approach
0U5F7ZZ	Destruction of Cul-de-sac, Via Natural or Artificial Opening
0U5F8ZZ	Destruction of Cul-de-sac, Via Natural or Artificial Opening Endoscopic
0UBF0ZZ	Excision of Cul-de-sac, Open Approach
0UBF3ZZ	Excision of Cul-de-sac, Percutaneous Approach
0UBF4ZZ	Excision of Cul-de-sac, Percutaneous Endoscopic Approach
0UBF7ZZ	Excision of Cul-de-sac, Via Natural or Artificial Opening
0UBF8ZZ	Excision of Cul-de-sac, Via Natural or Artificial Opening Endoscopic
<u>CPT Code</u>	
56303	Laparoscopy with excision of ovary or peritoneum
56307	Laparoscopic oophorectomy +/- salpingectomy
56308	Laparoscopy and vaginal hysterectomy +/- salpingo-oophorectomy
57531	Para-aortic lymph node sampling +/- salpingo-oophorectomy
58150	TAH +/- salpingo-oophorectomy
58152	TAH with colpo-urethrocystopexy +/- salpingo-oophorectomy
58180	Subtotal hysterectomy +/- salpingo-oophorectomy
58200	TAH with para-aortic and pelvic lymph node sampling +/- salpingo-oophorectomy

© 2022 Cham S et al. *JAMA Network Open.*

58210	Radical Hysterectomy
58240	Pelvic exenteration, including colostomy
58262	Vaginal Hysterectomy +/- salpingo-oophorectomy
58263	Vaginal Hysterectomy with repair of enterocele +/- salpingo-oophorectomy
58720	Salpingo-oophorectomy, complete or partial, unilateral or bilateral
58920	Wedge resection of ovary
58940	Oophorectomy, partial or total, unilateral or bilateral
58943	Oophorectomy, partial or total, unilateral or bilateral; for ovarian malignancy, with para-aortic and pelvic lymph node biopsies, peritoneal washings, peritoneal biopsies, diaphragmatic assessment, with or without salpingectomy(s), with or without omentectomy
58950	Resection of ovarian malignancy with bilateral salpingo-oophorectomy and omentectomy
58951	Resection of ovarian malignancy with bilateral salpingo-oophorectomy and omentectomy, with abdominal hysterectomy, pelvic and limited para-aortic lymphadenectomy)
58952	Resection of ovarian malignancy with bilateral salpingo-oophorectomy and omentectomy, with radical dissection for debulking
58953	Bilateral salpingo-oophorectomy with omentectomy, total abdominal hysterectomy and radical dissection for debulking
58954	Bilateral salpingo-oophorectomy with omentectomy, total abdominal hysterectomy and radical dissection for debulking, with pelvic lymphadenectomy and limited para-aortic lymphadenectomy)
58960	Laparotomy for staging or restaging of ovarian, tubal or primary peritoneal malignancy (second look) with or without omentectomy, peritoneal washing, biopsy of abdominal and pelvic peritoneum, diaphragmatic assessment with pelvic and limited per-aortic lymphadenectomy
**Biopsy codes**	
ICD-9 Codes	
54.24	percutaneous needle biopsy of abdominal mass
50.11	percutaneous needle biopsy of liver
34.91	Thoracentesis
40.1x-40.2	lymph node excision or biopsy
54.22	biopsy of abdominal wall/mass
54.23	biopsy of peritoneum
54.91	Percutaneous abdominal drainage
65.1x	operations on ovary (see above)
ICD-10 Codes	
0W9G30Z	Drainage of Peritoneal Cavity with Drainage Device, Percutaneous Approach
0W9G3ZX	Drainage of Peritoneal Cavity, Percutaneous Approach, Diagnostic

© 2022 Cham S et al. *JAMA Network Open.*

0W9G3ZZ	Drainage of Peritoneal Cavity, Percutaneous Approach
0FB03ZX	Excision of Liver, Percutaneous Approach, Diagnostic
0FB13ZX	Excision of Right Lobe Liver, Percutaneous Approach, Diagnostic
0FB23ZX	Excision of Left Lobe Liver, Percutaneous Approach, Diagnostic
0FD03ZX	Extraction of Liver, Percutaneous Approach, Diagnostic
0W993ZZ	Drainage of Right Pleural Cavity, Percutaneous Approach
0W9B3ZZ	Drainage of Left Pleural Cavity, Percutaneous Approach
07BH3ZZ	Excision of Right Inguinal Lymphatic, Percutaneous Approach
07BJ4ZZ	Excision of Left Inguinal Lymphatic, Percutaneous Endoscopic Approach
07BC3ZX	Excision of Pelvis Lymphatic, Percutaneous Approach, Diagnostic
07BC4ZX	Excision of Pelvis Lymphatic, Percutaneous Endoscopic Approach, Diagnostic
07BD3ZX	Excision of Aortic Lymphatic, Percutaneous Approach, Diagnostic
07BD4ZX	Excision of Aortic Lymphatic, Percutaneous Endoscopic Approach, Diagnostic
07DH3ZX	Extraction of Right Inguinal Lymphatic, Percutaneous Approach, Diagnostic
07DH4ZX	Extraction of Right Inguinal Lymphatic, Percutaneous Endoscopic Approach, Diagnostic
07DJ3ZX	Extraction of Left Inguinal Lymphatic, Percutaneous Approach, Diagnostic
07DJ4ZX	Extraction of Left Inguinal Lymphatic, Percutaneous Endoscopic Approach,
07DC3ZX	Extraction of Pelvis Lymphatic, Percutaneous Approach, Diagnostic
07DC4ZX	Extraction of Pelvis Lymphatic, Percutaneous Endoscopic Approach, Diagnostic
07DD3ZX	Extraction of Aortic Lymphatic, Percutaneous Approach, Diagnostic
07DD4ZX	Extraction of Aortic Lymphatic, Percutaneous Endoscopic Approach, Diagnostic
0WBF3ZX	Excision of Abdominal Wall, Percutaneous Approach, Diagnostic
0WBF4ZX	Excision of Abdominal Wall, Percutaneous Endoscopic Approach, Diagnostic
0WBFXZX	Excision of Abdominal Wall, External Approach, Diagnostic
0DBU3ZX	Excision of Omentum, Percutaneous Approach, Diagnostic
0DBU4ZX	Excision of Omentum, Percutaneous Endoscopic Approach, Diagnostic
0DBW3ZX	Excision of Peritoneum, Percutaneous Approach, Diagnostic
0DBW4ZX	Excision of Peritoneum, Percutaneous Endoscopic Approach, Diagnostic
0WBF0ZX	Excision of Abdominal Wall, Open Approach, Diagnostic
0WBF3ZX	Excision of Abdominal Wall, Percutaneous Approach, Diagnostic
0WBF4ZX	Excision of Abdominal Wall, Percutaneous Endoscopic Approach, Diagnostic
0UB04ZX	Excision of Right Ovary, Percutaneous Endoscopic Approach, Diagnostic
0UB08ZX	Excision of Right Ovary, Via Natural or Artificial Opening Endoscopic, Diagnostic
0UB14ZX	Excision of Left Ovary, Percutaneous Endoscopic Approach, Diagnostic
0UB18ZX	Excision of Left Ovary, Via Natural or Artificial Opening Endoscopic, Diagnostic
07TC0	Resection, lymphatic pelvis
07TD0	Resection, lymphatic aortic
-	

© 2022 Cham S et al. *JAMA Network Open.*

CPT codes	
10021-10022	FNA/biopsy procedures
76942	ultrasound guided procedure
32400	biopsy pleura percutaneous
47000-47001	incision/procedures of the liver
49082	Abdominal paracentesis (diagnostic or therapeutic) w/o imaging guidance
49083	Abdominal paracentesis (diagnostic or therapeutic) w/ imaging guidance
49180	Excision and Destruction Procedures on the Abdomen, Peritoneum, and Omentum
**Genetic Screening Codes**	
Gene specific codes (introduced in 2012, currently used)	
CPT Codes	Description
81162	BRCA1 (BRCA1, DNA repair associated), BRCA2 (BRCA2, DNA repair associated) (e.g., hereditary breast and ovarian cancer) gene analysis; full sequence analysis and full duplication/deletion analysis (i.e., detection of large gene rearrangements)
81163	BRCA1 (BRCA1, DNA repair associated), BRCA2 (BRCA2, DNA repair associated) (e.g., hereditary breast and ovarian cancer) gene analysis; full sequence analysis
81164	BRCA1 (BRCA1, DNA repair associated), BRCA2 (BRCA2, DNA repair associated) (e.g., hereditary breast and ovarian cancer) gene analysis; full duplication/deletion analysis (i.e., detection of large gene rearrangements)
81165	BRCA1 (BRCA1, DNA repair associated) (e.g., hereditary breast and ovarian cancer) gene analysis; full sequence analysis
81166	BRCA1 (BRCA1, DNA repair associated) (e.g., hereditary breast and ovarian cancer) gene analysis; full duplication/deletion analysis (i.e., detection of large gene rearrangements)
81167	BRCA2 (BRCA2, DNA repair associated) (e.g., hereditary breast and ovarian cancer) gene analysis; full duplication/deletion analysis (i.e., detection of large gene rearrangements)
81211	BRCA1, BRCA2 gene analysis; full sequence analysis and common duplication/deletion variants in BRCA1 (i.e., exon 13 del 3.835 kb, exon 13 dup 6 kb, exon 14-20 del 26 kb, exon 22 del 510 bp, exon 8-9 del 7.1 kb)
81212	BRCA1 (BRCA1, DNA repair associated), BRCA2 (BRCA2, DNA repair associated) (e.g., hereditary breast and ovarian cancer) gene analysis; 185delAG, 5385insC, 6174delT variants
81213	BRCA1, BRCA2 gene analysis; uncommon duplication/deletion variants
81214	BRCA1 gene analysis; full sequence analysis and common duplication/deletion variant

© 2022 Cham S et al. *JAMA Network Open.*

81215	BRCA1 (BRCA1, DNA repair associated) (e.g., hereditary breast and ovarian cancer) gene analysis; known familial variant
81216	BRCA2 (BRCA2, DNA repair associated) (e.g., hereditary breast and ovarian cancer) gene analysis; full sequence analysis
81217	BRCA2 (BRCA2, DNA repair associated) (e.g., hereditary breast and ovarian cancer) gene analysis; known familial variant
81432	Hereditary breast cancer-related disorders (e.g., hereditary breast cancer, hereditary ovarian cancer, hereditary endometrial cancer); genomic sequence analysis panel, must include sequencing of at least 14 genes, including ATM, BRCA1, BRCA2, BRIP1, CDH1, MLH1, MSH2, MSH6, NBN, PALB2, PTEN, RAD51C, STK11, and TP53
81433	Hereditary breast cancer-related disorders (e.g., hereditary breast cancer, hereditary ovarian cancer, hereditary endometrial cancer); duplication/deletion analysis panel, must include analyses for BRCA1, BRCA2, MLH1, MSH2, and STK11
Methodology based codes (until 2013)	
83891	Molecular diagnostics; isolation or extraction of highly purified nucleic acid, each nucleic acid type (i.e., DNA or RNA)
83898	Molecular diagnostics; amplification, target, each nucleic acid sequence
83904	Molecular diagnostics; mutation identification by sequencing, single segment, each segment
83909	Molecular diagnostics; separation and identification by high-resolution technique (e.g., capillary electrophoresis), each nucleic acid preparation
83912	Molecular diagnostics; interpretation and report
HCPCS S codes (until 2013)	
S3818	Complete gene sequence analysis; BRCA1 gene
S3819	Complete gene sequence analysis; BRCA2 gene
S3820	Complete BRCA1 and BRCA2 gene sequence analysis for susceptibility to breast and ovarian cancer
S3822	Single mutation analysis (in individual with a known BRCA1 or BRCA2 mutation in the family) for susceptibility to breast and ovarian cancer
S3823	Three-mutation BRCA1 and BRCA2 analysis for susceptibility to breast and ovarian cancer in Ashkenazi individuals

© 2022 Cham S et al. *JAMA Network Open.*
